# Prognostic significance of serum inflammation indexes in different Lauren classification of gastric cancer

**DOI:** 10.1002/cam4.3706

**Published:** 2021-01-06

**Authors:** Xin Yin, Tianyi Fang, Yimin Wang, Yufei Wang, Daoxu zhang, Chunfeng Li, Yingwei Xue

**Affiliations:** ^1^ Department of Gastroenterological Surgery Harbin Medical University Cancer Hospital Harbin Medical University Harbin China

**Keywords:** gastric cancer_1_, lauren classification_2_, nomogram_6_, platelet–lymphocyte ratio_4_, prognosis_5_, systemic immune‐inflammation index_3_

## Abstract

**Background:**

Inflammatory indexes are considered to be potential prognostic biomarkers for patients with gastric cancer (GC). However, little evidence has defined the prognostic significance of inflammatory indexes for GC with different Lauren classification.

**Methods:**

A total of 852 patients with GC were randomly selected consecutively into intestinal type and diffuse/mixed type groups. Group bias was reduced by propensity score matching. The cutoff values of inflammatory indexes were analyzed by receiver operating characteristic curve. The Kaplan–Meier method and log‐rank test were used to analyze the overall survival (OS). The chi‐square test was used to analyze the association between inflammatory indexes and clinical characteristics. The independent risk factor for prognosis in each group was analyzed by univariate and multivariate analyses based on logistic regression. The nomogram models were constructed by R studio.

**Results:**

Intestinal type GC patients (*p* < 0.05) had a lower percentage of neutrophils in stage I, higher percentage of neutrophils and higher platelet count in stage Ⅲ (*p* < 0.05). Systemic immune‐inflammation index (SII) (*p* < 0.001), pTNM stage (*p* < 0.001), and postoperative chemotherapy (*p* = 0.002) were independent risk factors for prognosis in the intestinal type group. Platelet–lymphocyte ratio (PLR) (*p* < 0.001) and pTNM stage (*p* = 0.001) were independent risk factors for prognosis in the diffuse/mixed type group. The area under the curve of the nomogram model in predicting 5‐year prognosis in the intestinal type group and diffuse/mixed type group were 0.807 and 0.788, respectively.

**Conclusion:**

SII combined with postoperative chemotherapy and pTNM stage were used to construct a nomogram model to predict the prognosis of intestinal type GC. PLR combined with pTNM stage can be used to construct a nomogram model for diffuse/mixed type GC patients.

## 1 INTRODUCTION

Gastric cancer (GC) is the sixth most common cancer and the third leading cause of cancer death, with more than 80,000 deaths every year.^1^ In order to better evaluate prognosis of patients according to the biological characteristics of cancer cells, a variety of classification methods have been proposed.^2^, ^3^ Since 1965, the Lauren classification according to the histological structure of cancer cells has been the most widely used clinical classification.^4^ It is generally believed that diffuse and mixed gastric cancer is easy to infiltrate the muscularis propria in the early stage and cause peritoneal metastasis, which leads to poor prognosis. However, intestinal type GC has a better susceptibility to adjuvant chemotherapy and mostly causes postoperative lymph node metastasis.^5^, ^6^, ^7^, ^8^ Current researches suggested that intestinal and diffuse GC not only have different pathogenesis, but also have great differences in tumor microenvironment. Li et al. ^9^ found that the density of CD8^+^ tumor‐infiltrating lymphocytes in diffuse GC was higher, but their function was significantly inhibited. However, Simon et al. ^10^ suggested that diffuse and mixed GC have fewer circulating natural killer (NK) cells and regulatory T cells. Although the results may differ due to the difference in study population or methods, it is worthwhile to develop simple, rapid, and reliable biomarkers to evaluate immune responses of patients with different Lauren classification, and further guide the clinical treatment and predict prognosis.

With the development and application of tumor immunity, the important role of immune cells in the progress and metastasis of malignant tumors has been gradually recognized, and it is expected to become a potential prognostic marker.^11^ From 2013 to 2018, Galon successfully applied the immune response in the tumor microenvironment to TNM‐I stage of colon cancer, which indicates that traditional staging systems combined with tumor immunity can provide clinicians with more useful treatment information.^12^, ^13^ However, due to the high heterogeneity of GC, immunohistochemical detection has limited clinical application. As a sensitive defense system, peripheral blood immune response is also an important part of tumor immunity. Our previous study has found that inflammatory indexes neutrophil–lymphocyte ratio (NLR) and platelet–lymphocyte ratio (PLR) were significantly better than traditional tumor markers in early diagnosis of GC.^14^ Lee et al. ^15^ showed that NLR, PLR, and changes in NLR or PLR were prognostic factor for overall survival (OS) in patients with advanced GC treated with oxaliplatin/5‐FU combination (FOLFOX) postoperative chemotherapy. Therefore, the significance of immune response characteristics of different patients according to Lauren classification is worthy of further study.

In this study, we retrospectively analyzed patients who underwent radical gastrectomy in the Harbin Medical University Cancer Hospital between April 2014 and November 2015. The expression of peripheral immune cells in different Lauren classification GC patients was analyzed. At the same time, the clinical applicability of different inflammatory indexes including NLR, PLR, and systemic immune‐inflammation index (SII) were further evaluated. Finally, we constructed predictive models by combining immunomarkers with clinicopathological features of patients based on Lauren classification.

## 2 MATERIALS AND METHODS

### 2.1 Patients

The diagnosis of GC was based on tissue samples obtained during gastroscopy and further confirmation by pathologists through examination of postoperative pathological tissue. During hospitalization, patients underwent routine preoperative examinations, including magnetic resonance imaging/gastric computed tomography (CT), abdominal ultrasonography, chest radiography, electrocardiography, hematological examination, and tumor marker examination. Some patients underwent positron emission tomography (PET)/CT if necessary.

Exclusion criteria were: (1) preoperative chemotherapy; (2) severe heart disease; (3) platelet therapy within 3 months before surgery; (4) steroid treatment during hospitalization; (5) active bleeding; (6) intravascular coagulation; (7) complication with abdominal infection or systemic infectious disease; and (8) hematological malignancy.

The operation standard is based on the Japanese Gastric Cancer Treatment Guidelines.^16^ All operations were performed frozen‐section examination to ensure that the margins were negative. All patients in the study achieved R0 resection, and all operations were performed by the chief physician. In order to control the quality of the operation, photos and tables were recorded for each operation. Sample of photographs and record tables are shown in Supplement 1.

Postoperative chemotherapy regimens were based on the National Comprehensive Cancer Network Clinical Practice Guidelines in Oncology.^17^ Oxaliplatin +capecitabine (XELOX) or oxaliplatin +S‐1 (SOX) are the main treatment options for patients with stage II or III GC. To ensure the accuracy of the study, we included 186 patients who received complete postoperative chemotherapy in our institution. We did not include patients who did not undergo treatment in our institution, or who returned to the local hospital after surgery without complete chemotherapy records.

According to the postoperative pathological report of Lauren classification, the mixed type had a dual pattern of glandular/solid (intestinal) and isolated‐cell carcinoma (diffuse). Based on the latest World Health Organization (WHO) classification, we classed patients with diffuse type and mixed type GC into the same group to analyze the biological characteristics of signet ring cell carcinoma. Therefore, all patients were divided into intestinal type and diffuse/mixed type groups. Clinicopathological data were saved in the Gastric Cancer Information Management System v1.2 of Harbin Medical University Cancer Hospital (Copyright No.2013SR087424, *http*:www.sgihmu.com), including sex, age, height, weight, tumor diameter, Borrmann type, tumor location, pTNM stage, hematological examination, tumor marker examination, histological type, tumor infiltration pattern (INF), vascular infiltration, nerve infiltration, and postoperative chemotherapy. pTNM stage is based on the Eighth American Joint Committee on Cancer Staging Manual edition.^2^ All patients were reexamined by tumor markers or radiology (ultrasound, CT, and gastroscopy) every 3–6 months. In addition, PET/CT examination was performed as needed.

### 2.2 Blood sample

Blood samples were collected from patients 1 week before surgery in fasting condition. A 2 ml of blood from the cubical vein were collected and sent to the blood laboratory where the serum was separated. For the calculation of the inflammatory index, neutrophil–lymphocyte ratio (NLR) = N/L, platelet–lymphocyte ratio (PLR) = P/L, systemic immune‐inflammation index (SII) = N × P/L (N = Neutrophil count, L = Lymphocyte count, and P = Platelet count).

### 2.3 Statistical analysis

To compare the differences in systemic inflammatory response between intestinal type and diffuse/mixed type, the time from surgery to death from any cause due to GC was calculated as the overall survival (OS). If patients were alive at the last follow‐up, they were censored. The 5‐year OS in the two groups was compared. Propensity score matching (PSM) was performed to minimize the influence of confounding factors on selection bias. The propensity scores were elicited from matched patients in a 1:1 ratio with greedy matching algorithms without replacement. All clinical and pathological characteristics, including sex, age, body mass index (BMI), tumor diameter, Borrmann type, tumor location, pTNM stage, and postoperative chemotherapy, were statistically analyzed to assess the imbalance before and after PSM.

The survival time was shown as median ±standard deviation. The diagnostic significance of each inflammatory index was calculated and compared with the receiver operating characteristic curve (ROC) analysis. The area under the curve (AUC) was calculated, and the optimal cutoff value of each inflammatory index was analyzed by the Youden index, which was calculated by the sensitivity− (1−specificity). The maximum value of the Youden index was the optimal cutoff value. The log‐rank test and Kaplan–Meier method were used to analyze survival curves. The chi‐square test was used to analyze the association between inflammatory index and clinicopathological factors. Univariate and multivariate analyses based on the logistic regression were used to analyze the independent risk factor for prognosis in each group. Odds ratios (ORs) and 95% confidence intervals (CIs) were estimated for each factor. The nomogram models were drawn through the R studio by “SvyNom” and “rms” packages.^18^ The relationship curve and scatter plot were drawn by GraphPad Prism 8. SPSS version 25.0 (SPSS Inc., Chicago, IL, USA) was used for analysis and *p* < 0.05 was considered statistically significant.

## 3 RESULTS

### 3.1 Clinical characteristics

A total of 852 patients were randomly selected. According to postoperative pathological reports, there were 469 and 383 patients in the intestinal type group and diffuse/mixed type group, respectively. The male: female ratio was 626: 226. The clinicopathological characteristics of the two groups are summarized in Table 1. Before PSM, the two groups had significant differences in age (*p* = 0.011). There were 256 (54.6%) and 242 (63.2%) patients aged ≤60 years in the intestinal type group and diffuse/mixed type group, respectively, and 213 (45.4%) and 141 (36.8%) patients aged >60 years. After PSM, the two groups were matched 1: 1, with 378 patients in each group. Each variable was well balanced without significant differences (All *p* > 0.05) (Table 1).

**TABLE 1:** Baseline characteristics of the patients before and after PSM.

Characteristics	Before PSM			After PSM		
	Intestinal type (469)	Diffuse type and Mixed type (383)	*P* value	Intestinal type (378)	Diffuse type and Mixed type (378)	*P* value
Sex			0.595			0.566
Female	121 (25.8)	105 (27.4)		280 (74.1)	273 (72.2)	
Male	348 (74.2)	278 (72.6)		98 (25.9)	105 (27.8)	
Age (years)			**0.011**			0.332
≤60	256 (54.6)	242 (63.2)		224 (59.3)	237 (62.7)	
>60	213 (45.4)	141 (36.8)		154 (40.7)	141 (37.3)	
BMI			0.133			0.167
≤22.59	228 (48.6)	206 (53.8)		186 (49.2)	205 (54.2)	
>22.59	241 (51.4)	177 (46.2)		192 (50.8)	173 (45.8)	
Borrmann type			0.098			0.157
0–2	204 (43.5)	155 (40.5)		165 (43.7)	153 (40.5)	
3	226 (48.2)	179 (46.7)		181 (47.9)	177 (46.8)	
4	39 (8.3)	49 (12.8)		32 (8.5)	48 (12.7)	
Tumor diameter (mm)			0.955			0.708
≤50	296 (63.1)	241 (62.9)		232 (61.4)	237 (62.7)	
>50	173 (36.9)	142 (37.1)		146 (38.6)	141 (37.3)	
Tumor location			0.191			0.200
Middle and Upper third	94 (20.0)	85 (22.2)		71 (18.8)	85 (22.5)	
Lower third	330 (70.4)	249 (65.0)		268 (70.9)	245 (64.8)	
Entire stomach	45 (9.6)	49 (12.8)		39 (10.3)	48 (12.7)	
pTNM stage			0.230			0.262
Ⅰ	137 (29.2)	104 (27.2)		112 (29.6)	100 (26.5)	
Ⅱ	158 (33.7)	115 (30.0)		124 (32.8)	114 (30.2)	
Ⅲ	174 (37.1)	164 (42.8)		142 (37.6)	164 (43.4)	
Postoperative chemotherapy			0.344			0.531
Yes	151 (32.2)	115 (30.0)		123 (32.5)	115 (30.4)	
No	318 (67.8)	268 (70.0)		255 (67.5)	263 (69.6)	

BMI: body mass index.

Statistically significant *P* values are in bold (*p* < 0.05).

### 3.2 Survival based on Lauren classification

**FIGURE 1:**
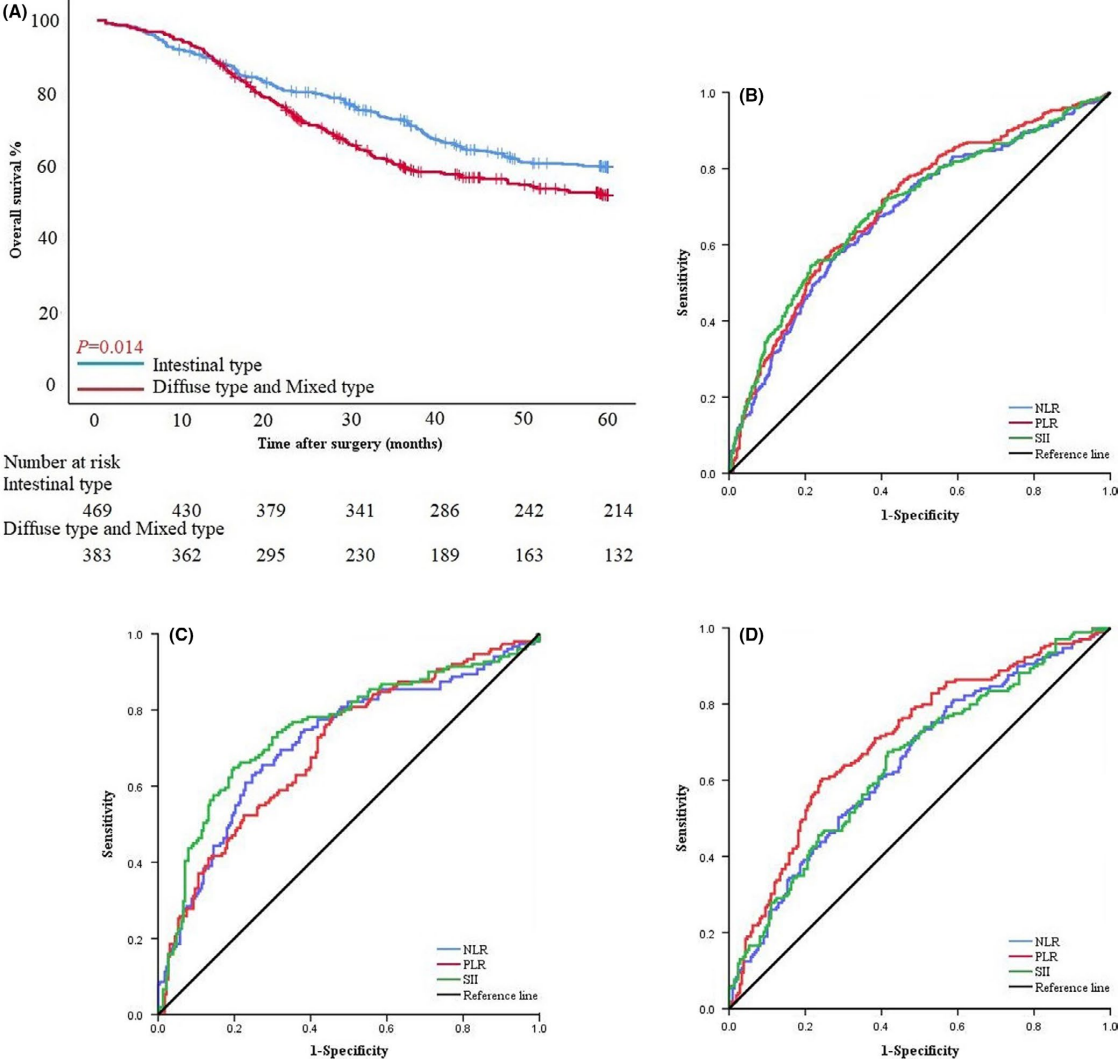
(A) Survival curves based on patients with intestinal type and diffuse/mixed type before PSM. (B) ROC curve of NLR, PLR, and SII among all patients in PSM cohort. (C) ROC curve of NLR, PLR, and SII of patients with intestinal type. (D) ROC curve of NLR, PLR, and SII of patients with diffuse/mixed type.

Before PSM, patients in the intestinal type group had better OS than those in the diffuse/mixed type group. The survival time of patients with intestinal type was 57.13 ± 19.48 months and 5‐year survival rate was 45.6%, and the survival time of patients with diffuse/mixed type was 37.57 ± 19.52 months and 5‐years survival rate was 34.5% (*p* = 0.014; HR 1.298, 95% CI 1.053–1.600) (Figure 1A).

### 3.3 NLR, PLR, and SII score

NLR, PLR, and SII score of 1.99, 126.90, and 529.24, respectively, were calculated as the most appropriate cutoff thresholds by the Youden index of the ROC curve for all patients after PSM based on preoperative hematology. The AUC were 0.679 (95% CI: 0.640–0.718), 0.702 (95% CI: 0.664–0.740), and 0.697 (95% CI: 0.658–0.735), respectively (Figure 1B). The AUC of NLR, PLR, and SII were 0.716 (95% CI: 0.661–0.770), 0.701 (95% CI: 0.647–0.755), and 0.750 (95% CI: 0.698–0.803), respectively, in the intestinal type group, and 0.646 (95% CI: 0.590–0.701), 0.707 (95% CI: 0.654–0.759), and 0.647 (95% CI: 0.592–0.703) in the diffuse/mixed type group (Figure 1C and D).

### 3.4 Connection between inflammatory index and basic clinicopathological characteristics

**FIGURE 2:**
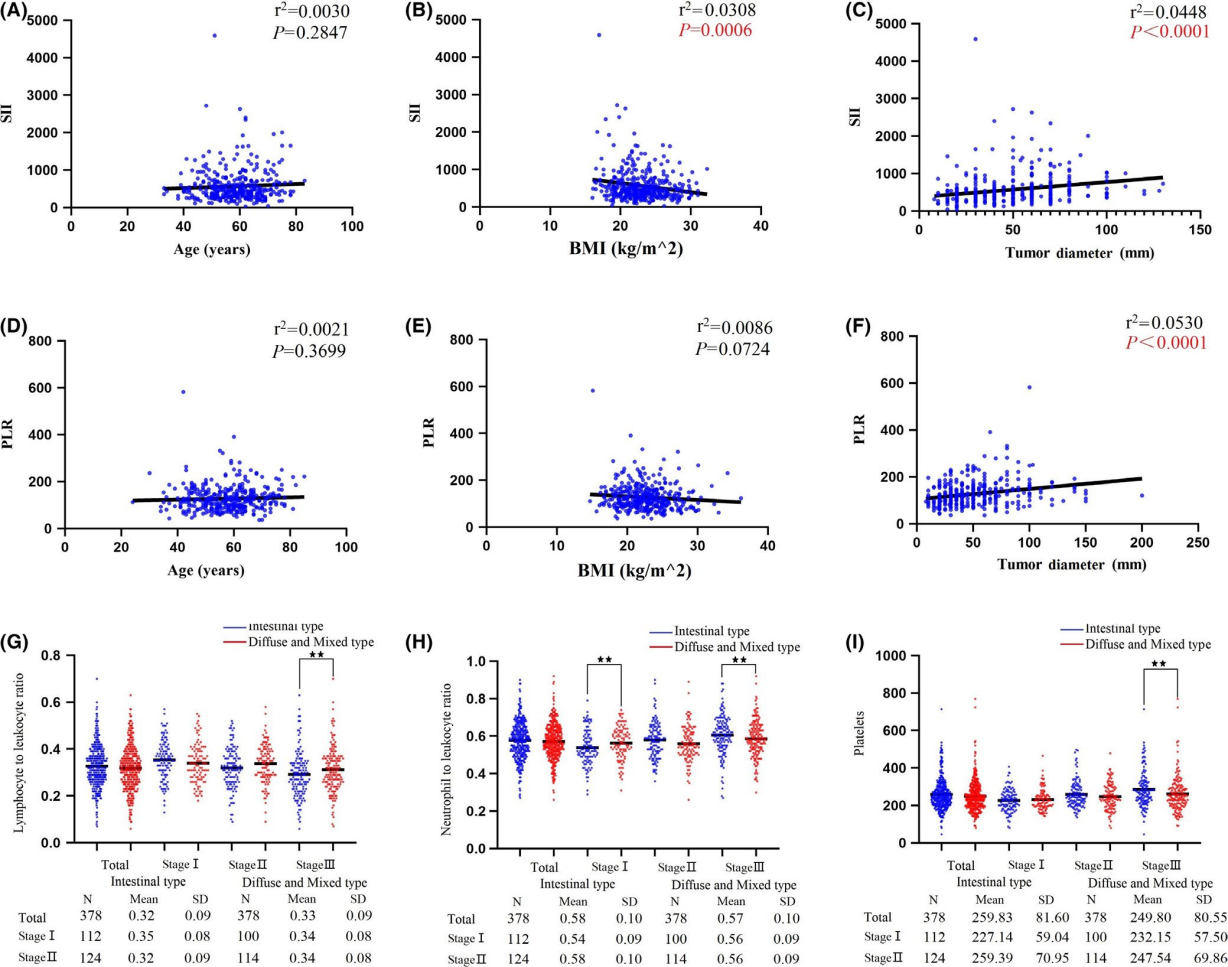
(A–C) Connection between SII and age, BMI, and tumor diameter in patients with intestinal type. (D, E) Connection between PLR and age, BMI, and tumor diameter in patients with diffuse/mixed type. (G–I) The differences in lymphocyte to leukocyte ratio, neutrophil to leukocyte ratio and platelet count between intestinal type group and diffuse/mixed type group based on pTNM stage (★★: *p* < 0.05).

In the PSM cohort of the intestinal type group, SII was negatively correlated with BMI (r^2^=0.0308, *p* = 0.0006), positively correlated with tumor diameter (r^2^=0.0448, *p* < 0.0001) but had no correlation with age (r^2^=0.0030, *p* = 0.2847) (Figure 2A–C). In the diffuse/mixed type group, PLR was positively correlated with tumor diameter (r^2^=0.0530, *p* < 0.0001) but had no correlation with age and BMI (r^2^=0.0021, *p* = 0.3699. r^2^=0.0086, *p* = 0.0724) (Figure 2D–F).

Peripheral circulating immune cells, including the percentage of neutrophils and lymphocytes and platelet count were analyzed according to pTNM stage. For patients with stage I GC, the percentage of neutrophils in the intestinal type group was lower (*p* < 0.05), and the percentage of neutrophils was higher in stage Ⅲ GC (*p* < 0.05). The percentage of lymphocytes was the opposite in stage Ⅲ GC. Platelet count was higher in the intestinal group among patients with stage Ⅲ GC (Figure 2G–I).

### 3.5 Inflammatory index and patient survival

**FIGURE 3:**
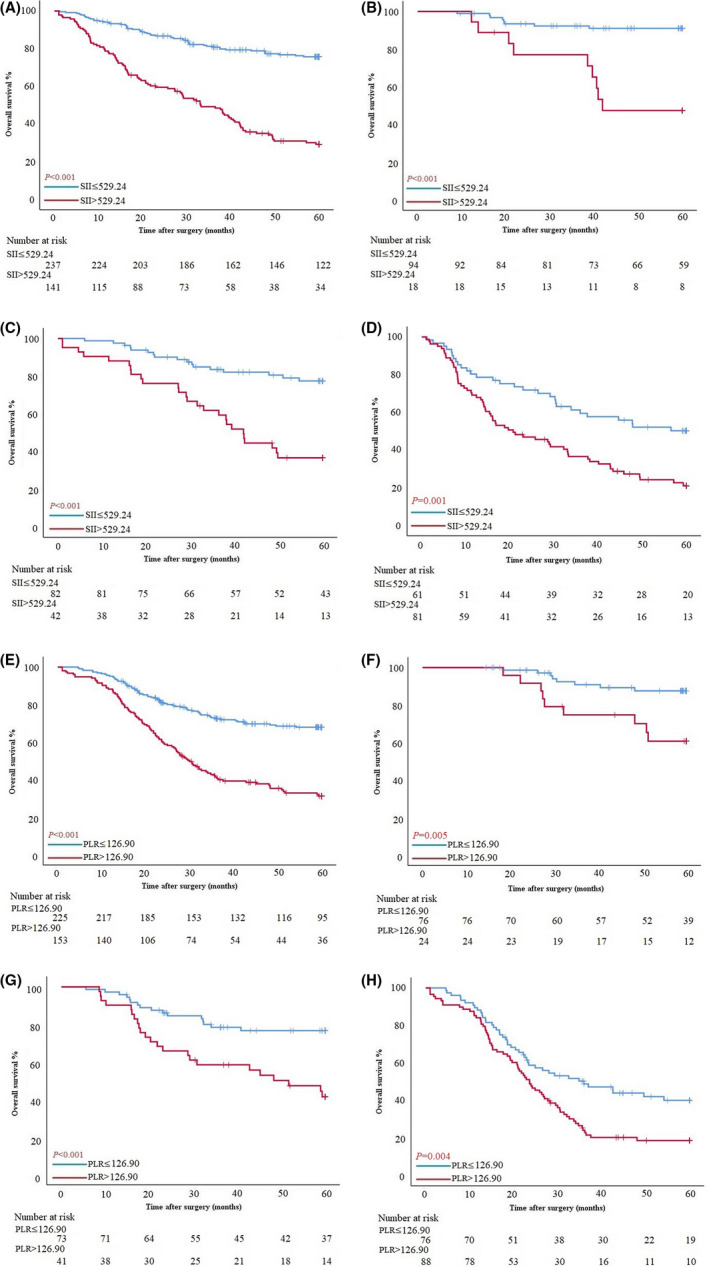
**(**A–D) Survival curves of patients with intestinal type based on SII in all stages, stage Ⅰ, stage Ⅱ, and stage Ⅲ. (E–H) Survival curves of patients with diffuse/mixed type based on PLR in all stages, stage Ⅰ, stage Ⅱ, and stage Ⅲ.

Patients with intestinal type in the PSM cohort had a significant difference in OS between SII>529.60 and SII≤529.60 (OS: 60.00 ± 18.24 months vs. 32.20 ± 20.59 months, *p* < 0.001; HR 4.187, 95% CI 2.997–5.851). According to the pTNM stage, in stage Ⅰ, Ⅱ, and Ⅲ, patients with SII≤529.60 all had better survival (OS: 60.00 ± 15.56 months vs. 41.43 ± 18.41 months, *p* < 0.001; HR 6.724, 95% CI 2.590–17.456. 60.00 ± 16.23 months vs. 40.74 ± 19.26 months, *p* < 0.001; HR 3.707, 95% CI 2.008–6.845. 45.90 ± 24.93 months vs. 20.73 ± 20.29 months, *p* = 0.001; HR 2.155, 95% CI 1.382–3.358) (Figure 3A–D). SII score had a significant association with sex, BMI, NLR, PLR, carcinoembryonic antigen (CEA), Borrmann type, tumor diameter, pTNM stage, vascular infiltration, and nerve infiltration by chi‐square test in clinical and pathological features (*p* = 0.040, *p* = 0.007, *p* < 0.001, *p* < 0.001, *p* = 0.017, *p* < 0.001, *p* < 0.001, *p* < 0.001, *p* = 0.029, and *p* = 0.022) (Table 2).

**TABLE 2:** The chi‐square test analysis of the connection between inflammation index and clinicopathological features.

Characteristics	Intestinal type			Diffuse type and Mixed type		
	SII≤529.24 (237)	SII>529.24 (141)	*P* value	PLR≤126.90 (225)	PLR>126.90 (153)	*P* value
Sex			**0.040**			0.559
Male	184 (77.6)	96 (68.1)		165 (73.3)	108 (70.6)	
Female	53 (22.4)	45 (31.9)		60 (26.7)	45 (29.4)	
Age (years)			0.229			**0.031**
≤60	146 (61.6)	78 (55.3)		151 (67.1)	86 (56.2)	
>60	91 (38.4)	63 (44.7)		74 (32.9)	67 (43.8)	
BMI			**0.007**			0.205
≤22.59	104 (43.9)	82 (58.2)		116 (51.6)	89 (58.2)	
>22.59	133 (56.1)	59 (41.8)		109 (48.4)	64 (41.8)	
NLR			**<0.001**			**<0.001**
≤1.99	203 (85.7)	21 (14.9)		164 (72.9)	56 (36.6)	
>1.99	34 (14.3)	120 (85.1)		61 (27.1)	97 (63.4)	
PLR			**<0.001**	‐	‐	‐
≤126.90	195 (82.3)	27 (19.1)				
>126.90	42 (17.7)	114 (80.9)				
SII	‐	‐	‐			**<0.001**
≤529.24				191 (84.9)	60 (39.2)	
>529.24				34 (15.1)	93 (60.8)	
CEA			**0.017**			0.345
≤5 ng/ml	207 (87.3)	110 (78.0)		200 (88.9)	131 (85.6)	
>5 ng/ml	30 (12.7)	31 (22.0)		25 (11.1)	22 (14.4)	
CA19‐9			0.071			**0.028**
≤37 U/ml	205 (86.5)	112 (79.4)		205 (91.1)	128 (83.7)	
>37 U/ml	32 (13.5)	29 (20.6)		20 (8.9)	25 (16.3)	
CA72‐4			0.699			0.075
≤15.520 U/ml	214 (90.3)	129 (91.5)		201 (89.3)	127 (83.0)	
>15.520 U/ml	23 (9.7)	12 (8.5)		24 (10.7)	26 (17.0)	
Borrmann type			**<0.001**			0.080
0–2	130 (54.9)	35 (24.8)		98 (43.6)	55 (35.9)	
3	90 (38.0)	91 (64.5)		105 (46.7)	72 (47.1)	
4	17 (7.2)	15 (10.6)		22 (9.8)	26 (17.0)	
Tumor diameter (mm)			**<0.001**			**<0.001**
≤50	171 (72.2)	61 (43.3)		162 (72.0)	75 (49.0)	
>50	66 (27.8)	80 (56.7)		63 (28.0)	78 (51.0)	
Tumor location			0.061			**0.008**
Middle and Upper third	39 (16.5)	32 (22.7)		56 (24.9)	29 (19.0)	
Lower third	178 (75.1)	90 (63.8)		150 (66.7)	95 (62.1)	
Entire stomach	20 (8.4)	19 (13.5)		19 (8.4)	29 (19.0)	
pTNM stage			**<0.001**			**<0.001**
Ⅰ	94 (39.7)	18 (12.8)		76 (33.8)	24 (15.7)	
Ⅱ	82 (34.6)	42 (29.8)		73 (32.4)	41 (26.8)	
Ⅲ	61 (25.7)	81 (57.4)		76 (33.8)	88 (57.5)	
Tumor infiltration pattern (INF)			0.761			0.966
INFa	121 (51.1)	73 (51.8)		43 (19.1)	28 (18.3)	
INFb	83 (35.0)	52 (36.9)		58 (25.8)	41 (26.8)	
INFc	33 (13.9)	16 (11.3)		124 (55.1)	84 (54.9)	
Vascular infiltration			**0.029**			**0.008**
No	161 (67.9)	80 (56.7)		159 (70.7)	88 (57.5)	
Yes	76 (32.1)	61 (43.3)		66 (29.3)	65 (42.5)	
Nerve infiltration			**0.022**			0.186
No	133 (56.1)	62 (44.0)		120 (53.3)	71 (46.4)	
Yes	104 (43.9)	79 (56.0)		105 (46.7)	82 (53.6)	

BMI: body mass index, NLR: neutrophil–lymphocyte ratio, PLR: platelet–lymphocyte ratio, SII: systemic immune‐inflammation index, CEA: carcinoembryonic antigen, CA19‐9: carbohydrate antigen 19–9, CA72‐4: carbohydrate antigen 72–4.

CEA, CA19‐9, and CA72‐4 were according to the tumor marker examination. Tumor location, tumor infiltration pattern, vascular infiltration, and nerve infiltration were according to the postoperative pathology report. INFa: expanding growth and a distinct border with the surrounding tissue, INFc: infiltrating growth and an indistinct border with the surrounding tissue, INFb: in‐between INFa and INFc. Statistically significant *P* values are in bold (*p* < 0.05).

Patients with diffuse/mixed type in the PSM cohort had a significant difference in OS between PLR>127.56 and PLR≤127.56 (OS: 53.87 ± 18.83 months vs. 29.20 ± 19.15 months, *p* < 0.001; HR 2.824, 95% CI 2.073–3.847). According to the pTNM stage, in stage Ⅰ, Ⅱ, and Ⅲ, patients with PLR≤127.56 all had better survival (OS: 60.00 ± 15.52 months vs. 59.75 ± 15.09 months, *p* = 0.005; HR 3.623, 95% CI 1.397–9.394. 60.00 ± 17.99 months vs. 42.80 ± 19.74 months, *p* < 0.001; HR 2.950, 95% CI 1.557–5.587. 29.90 ± 19.22 months vs. 23.62 ± 16.53 months, *p* = 0.004; HR 1.748, 95% CI 1.193–2.561) (Figure 3E–H). PLR score had a significant association with age, NLR, SII, carbohydrate antigen (CA)19–9, tumor diameter, tumor location, pTNM stage, and vascular infiltration by chi‐square test in clinical and pathological features (*p* = 0.031, *p* < 0.001, *p* < 0.001, *p* = 0.028, *p* < 0.001, *p* = 0.008, *p* < 0.001, and *p* = 0.008) (Table 2).

### 3.6 Inflammatory index and postoperative chemotherapy

**FIGURE 4:**
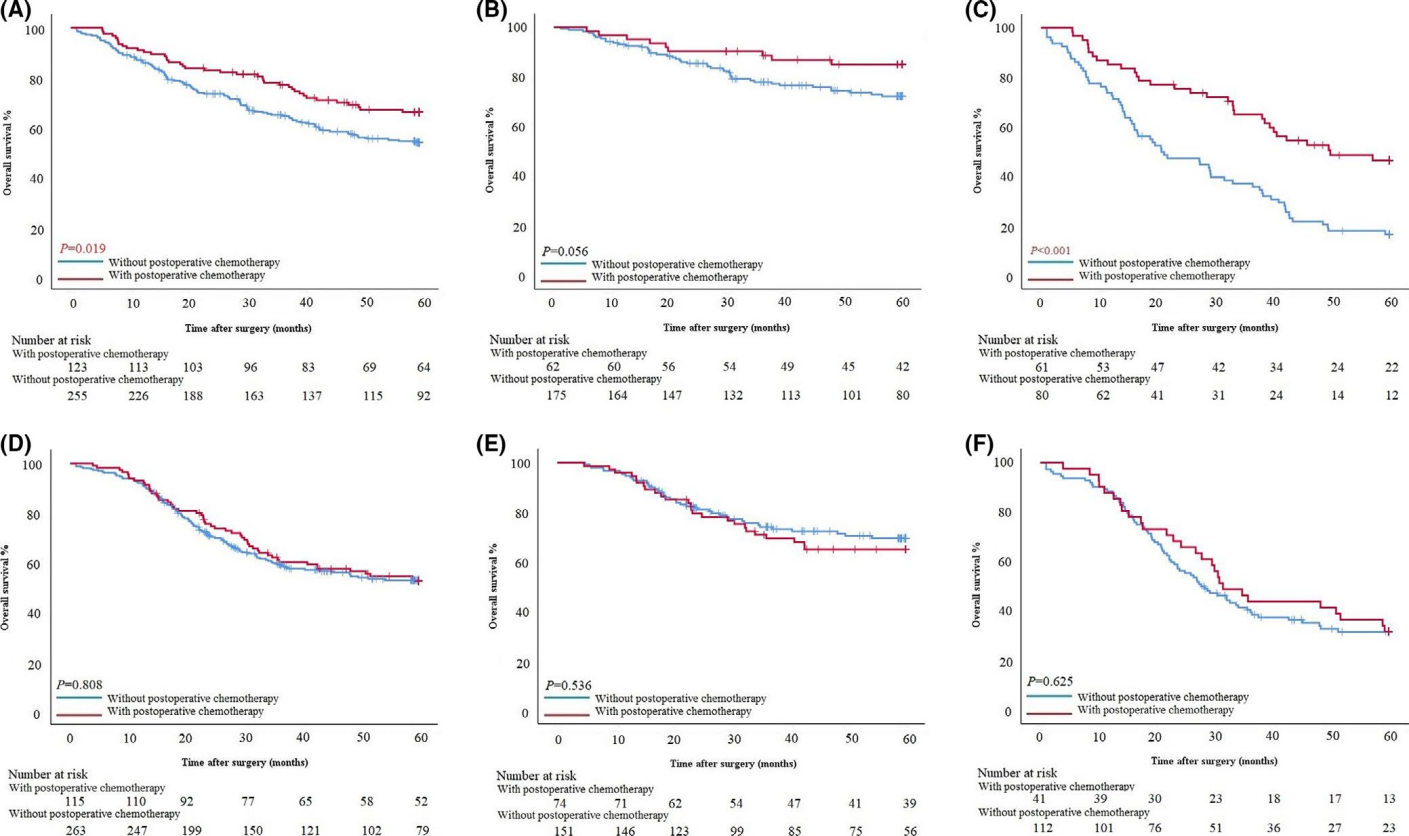
**(**A–C) Survival curves based on with and without postoperative chemotherapy in all, SII≤529.60 and SII>529.60 intestinal type GC patients. (D–F) Survival curves based on with and without postoperative chemotherapy in all, PLR≤126.90 and PLR>126.90 diffuse/mixed type GC patients.

Patients with intestinal type in the PSM cohort with postoperative chemotherapy had better survival (*p* = 0.019). The difference was not found in patients with SII≤529.60 (*p* = 0.056), but in patients with SII>529.60 (OS: 44.33 ± 19.56 months vs. 20.70 ± 19.74 months, *p* < 0.001; HR 1.077, 95% CI 0.622–1.865) (Figure 4A–C). On the contrary, in the diffuse/mixed type group, there was no significant difference in survival between patients with and without postoperative chemotherapy (*p* = 0.808, *p* = 0.536, and *p* = 0.625) (Figure 4D–F).

### 3.7 Univariate and multivariate regression analyses in two groups

To identify the independent risk factors for prognosis in the two groups, univariate and multivariate analyses based on the logistic risk regression model were implemented. According to univariate analysis, age (*p* < 0.001, *p* = 0.021), CA19‐9 (*p* = 0.001, *p* = 0.003), Borrmann type (*p* < 0.001, *p* < 0.001), tumor diameter (*p* < 0.001, *p* < 0.001), tumor location (*p* < 0.001, *p* = 0.010), pTNM stage (*p* < 0.001, *p* < 0.001), tumor infiltration pattern (*p* = 0.017, *p* = 0.045), vascular infiltration (*p* = 0.001, *p* < 0.001), and nerve infiltration (*p* < 0.001, *p* < 0.001). SII (*p* < 0.001) and postoperative chemotherapy (*p* = 0.041) were significant in the intestinal type group, PLR (*p* < 0.001) and CEA (*p* = 0.033) were significant in the diffuse/mixed type group. According to multivariate analyses, SII (*p* < 0.001), pTNM stage (*p* < 0.001), and postoperative chemotherapy (*p* = 0.002) were independent risk factors for prognosis in the intestinal type group, and PLR (*p* < 0.001) and pTNM stage (*p* = 0.001) were independent risk factors for prognosis in the diffuse/mixed type group (Tables 3 and 4).

**TABLE 3:** Prognosis factors of patients with GC by univariate and multivariate based on logistic regression analysis in intestinal type group.

Characteristics	Intestinal type			
	Univariate analysis		Multivariate analysis	
	OR (95% CI)	*P* value	OR (95% CI)	*P* value
Sex		0.061	—	—
Male	1			
Female	1.560 (0.980–2.482)			
Age (years)	1.051 (1.026–1.077)	**<0.001**	1.024 (0.994–1.056)	0.120
BMI	0.974 (0.913–1.038)	0.416	—	—
SII	1.002 (1.002–1.003)	**<0.001**	1.002 (1.001–1.003)	**<0.001**
CEA	1.019 (1.000–1.038)	0.055	—	—
CA19‐9	1.004 (1.002–1.006)	**0.001**	1.002 (0.999–1.004)	0.138
CA72‐4	1.004 (0.996–1.011)	0.324	—	—
Borrmann type		**<0.001**		0.168
0–2	1		1	
3	2.771 (1.757–4.371)	**<0.001**	1.518 (0.851–2.707)	0.158
4	5.591 (2.489–12.558)	**<0.001**	2.920 (0.790–10.793)	0.108
Tumor diameter (mm)	1.024 (1.014–1.034)	**<0.001**	0.994 (0.980–1.007)	0.354
Tumor location		**<0.001**		0.510
Middle and Upper third	1		1	
Lower third	0.492 (0.289–0.835)	**0.009**	0.709 (0.371–1.355)	0.299
Entire stomach	1.736 (0.778–3.875)	0.178	1.081 (0.312–3.740)	0.903
pTNM stage		**<0.001**		**<0.001**
Ⅰ	1		1	
Ⅱ	2.967 (1.572–5.598)	**0.001**	2.345 (1.030–5.336)	**0.042**
Ⅲ	9.971 (5.366–18.529)	**<0.001**	6.607 (2.703–16.147)	**<0.001**
Tumor infiltration pattern (INF)		**0.017**		0.299
INFa	1		1	
INFb	1.886 (1.201–2.962)	**0.006**	1.550 (0.879–2.733)	0.130
INFc	1.655 (0.875–3.131)	0.121	1.077 (0.479–2.421)	0.858
Vascular infiltration		**0.001**		0.703
No	1		1	
Yes	2.064 (1.344–3.168)		0.894 (0.501–1.594)	
Nerve infiltration		**<0.001**		0.949
No	1		1	
Yes	2.441 (1.600–3.723)		1.020 (0.554–1.878)	
Postoperative chemotherapy		**0.041**		**0.002**
Yes	1		1	
No	0.625 (0.398–0.982)		0.387 (0.211–0.710)	

BMI: body mass index, SII: systemic immune‐inflammation index, CEA: carcinoembryonic antigen, CA19‐9: carbohydrate antigen 19–9, CA72‐4: carbohydrate antigen 72–4.

CEA, CA19‐9, and CA72‐4 were according to the tumor marker examination. Tumor location, tumor infiltration pattern, vascular infiltration, and nerve infiltration were according to the postoperative pathology report. INFa: expanding growth and a distinct border with the surrounding tissue, INFc: infiltrating growth and an indistinct border with the surrounding tissue, INFb: in‐between INFa and INFc. Statistically significant *P* values are in bold (*p* < 0.05).

**TABLE 4:** Prognosis factors of patients with GC by univariate and multivariate based on logistic regression analysis in diffuse type and mixed type group.

Characteristics	Diffuse type and Mixed type			
	Univariate analysis		Multivariate analysis	
	OR (95% CI)	*P* value	OR (95% CI)	*P* value
Sex		0.497	‐	‐
Male	1			
Female	0.854 (0.542–1.346)			
Age (years)	1.024 (1.004–1.045)	**0.021**	1.024 (0.998–1.050)	0.067
BMI	0.994 (0.935–1.057)	0.848	‐	‐
PLR	1.014 (1.009–1.019)	**<0.001**	1.011 (1.005–1.016)	**<0.001**
CEA	1.040 (1.003–1.079)	**0.033**	1.013 (0.984–1.043)	0.371
CA19‐9	1.010 (1.003–1.017)	**0.003**	1.006 (1.000–1.012)	0.053
CA72‐4	1.005 (0.997–1.013)	0.231	‐	‐
Borrmann type		**<0.001**		0.865
0–2	1		1	
3	2.196 (1.401–3.442)	**0.001**	1.172 (0.651–2.108)	0.597
4	3.537 (1.800–6.954)	**<0.001**	1.088 (0.252–4.691)	0.910
Tumor diameter (mm)	1.024 (1.015–1.032)	**<0.001**	1.002 (0.990–1.014)	0.726
Tumor location		**0.010**		0.564
Middle and Upper third	1		1	
Lower third	0.800 (0.486–1.315)	0.378	0.772 (0.413–1.443)	0.417
Entire stomach	2.151 (1.037–4.460)	**0.040**	1.333 (0.364–4.879)	0.664
pTNM stage		**<0.001**		**0.001**
Ⅰ	1		1	
Ⅱ	2.539 (1.326–4.861)	**0.005**	1.377 (0.644–2.941)	0.409
Ⅲ	10.818 (5.833–20.063)	**<0.001**	3.847 (1.695–8.729)	**0.001**
Tumor infiltration pattern (INF)		0.124		0.967
INFa	1		1	
INFb	1.700 (0.905–3.194)	0.099	1.016 (0.469–2.199)	0.968
INFc	1.779 (1.014–3.120)	**0.045**	0.943 (0.468–1.898)	0.869
Vascular infiltration		**<0.001**		0.487
No	1		1	
Yes	2.927 (1.888–4.537)		1.226 (0.691–2.177)	
Nerve infiltration		**<0.001**		0.055
No	1		1	
Yes	3.196 (2.093–4.880)		1.766 (0.989–3.153)	
Postoperative chemotherapy		0.722	‐	‐
Yes	1			
No	1.083 (0.698–1.682)			

BMI: body mass index, PLR: platelet–lymphocyte ratio, CEA: carcinoembryonic antigen, CA19‐9: carbohydrate antigen 19–9, CA72‐4: carbohydrate antigen 72–4.

CEA, CA19‐9, and CA72‐4 were according to the tumor marker examination. Tumor location, tumor infiltration pattern, vascular infiltration, and nerve infiltration were according to the postoperative pathology report. INFa: expanding growth and a distinct border with the surrounding tissue, INFc: infiltrating growth and an indistinct border with the surrounding tissue, INFb: in‐between INFa and INFc. Statistically significant *P* values are in bold (*p* < 0.05).

### 3.8 Nomogram

**FIGURE 5:**
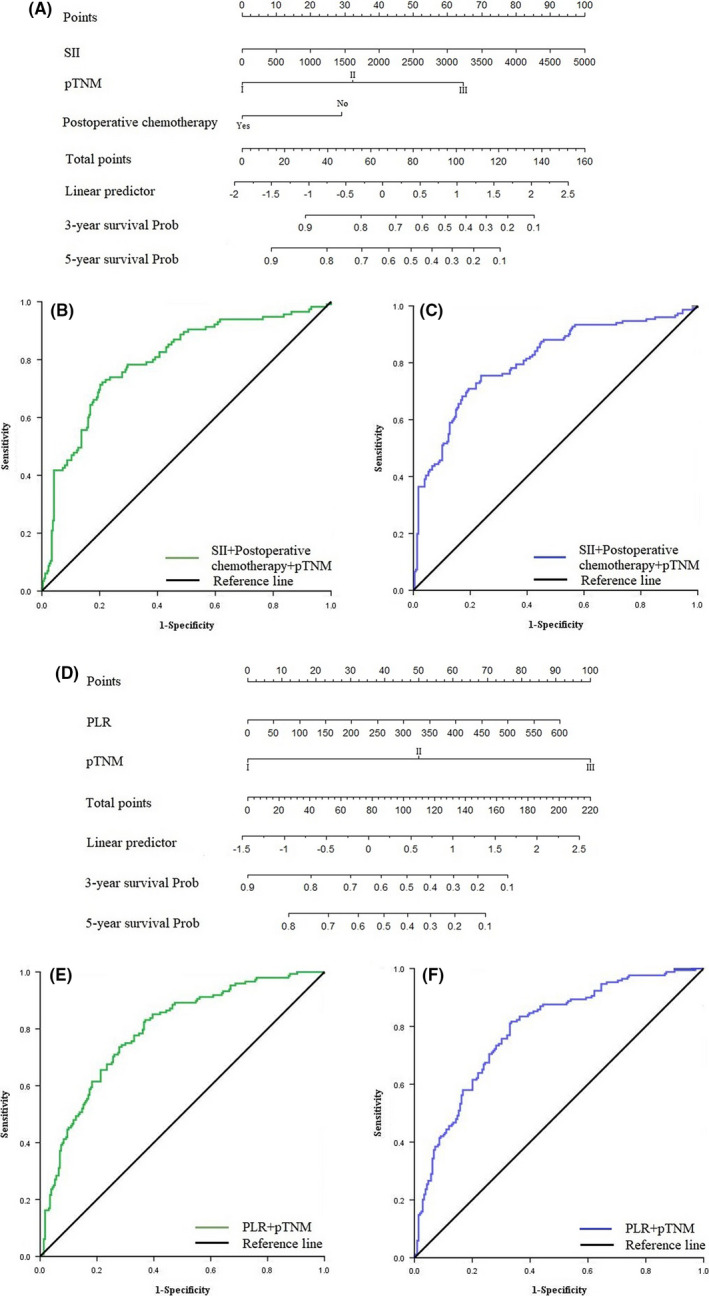
(A) Nomogram model predicting 3‐ and 5‐year survival of patients with intestinal type GC. (B) ROC curve of nomogram model predicting 3‐year survival of patients with intestinal type GC. (C) ROC curve of nomogram model predicting 5‐year survival of patients with intestinal type GC. (D) Nomogram model predicting 3‐ and 5‐year survival of patients with diffuse/mixed type GC. (E) ROC curve of nomogram model predicting 3‐year survival of patients with diffuse/mixed type GC. (F) ROC curve of nomogram model predicting 5‐year survival of patients with diffuse/mixed type GC.

Nomogram models in predicting the prognosis of patients were constructed for different Lauren classification groups (Figure 5A and D). The AUC of the model in predicting prognosis within 3 and 5 years were 0.796 (95% CI: 0.745–0.847) and 0.807 (95% CI: 0.761–0.853) in the intestinal type group. The sensitivity were 72.2% and 75.5%, respectively, and the specificity were 79.1% and 76.2%, respectively (Figure 5B and C). The AUC of the model in predicting prognosis within 3 and 5 years were 0.791 (95% CI: 0.745–0.837) and 0.788 (95% CI: 0.743–0.834) in the diffuse/mixed type group. The sensitivity were 83.1% and 81.7%, respectively, and the specificity were 63.0% and 66.5%, respectively (Figure 5E and F).

## 4 DISCUSSION

Over the past 50 years, researchers have evaluated the incidence of GC, location of the tumor and response to postoperative chemotherapy more accurately due to the popularity of Lauren classification. For example, intestinal type GC is mainly distributed in the Asian population, and it is more sensitive to targeted therapy and adjuvant chemotherapy, which provides a powerful help for individualized treatment.^19^, ^20^ With the breakthrough of tumor immunotherapy, many studies have found that the immune response of the tumor microenvironment and peripheral blood of patients with different Lauren classification differ. The purpose of this study was to investigate the significance of peripheral blood immune response in evaluating the prognosis and individualized chemotherapy in patients with different Lauren type GC.

At present, it is believed that diffuse and intestinal types GC have different oncological characteristics. Intestinal type GC is mainly caused by progression of chronic inflammation into atrophic and metaplastic gastritis and Helicobacter pylori infection. Microscopically, the structure is mostly gland lumen which composed by larger and more morphologically variable cancer cells.^4^, ^21^, ^22^, ^23^ Diffuse GC is mostly caused by active inflammation of the gastric mucosa, and the pathological manifestation is scattered isolated cells or small cell clusters. This particular structure is closely related to the release of interleukin (IL)‐6, IL‐8, and transforming growth factor (TGF)‐β1 in the progression of epithelial–mesenchymal transition (EMT), which is also symbolic biological behavior of diffuse type GC.^24^, ^25^, ^26^ These different oncological characteristics may be related to the following mechanisms. First, diffuse GC has a low mutation, diffuse tissue structure, without programed death ligand 1 expression. Second, CDH1 mutation of E‐cadherin gene is one of the representative variants of diffuse GC, and is closely related to the process of genetic EMT.^23^, ^27^, ^28^ The RhoA pathway plays an important role in proliferation of diffuse GC.^29^ These complex mechanisms can be associated with a variety of cytokines, such as IL‐6 and Sox2, which give diffuse GC a unique tumor immune environment. According to recent studies, CD8+ T cells in diffuse GC have high density, but are inhibited by IL‐10, TGF‐β, and indoleamine 2,3‐dioxygenase 1. In addition, there were lower levels of NK cells and regulatory T cells in the peripheral blood of patients with advanced diffuse GC, while there was no significant difference in the levels of CD4+ T cells, CD8+ T cells, and myeloid derived suppressor cells. These differences may lead to the strong distant metastasis of diffuse GC.^9^, ^10^

In this study, we found that although there was no significant difference in the distribution of immune cells in peripheral blood of patients with intestinal type and diffuse/mixed type GC, stage I intestinal type patients had a lower percentage of neutrophils. Stage III intestinal type patients had a higher percentage of neutrophils, platelet count, and lower percentage of lymphocytes. Matowicka Karna et al. ^30^ found that expression of IL‐6 in early GC tissue was significantly increased, which suggested that the tumor immune microenvironment in the early stage was mainly caused by local acute inflammation. The proliferation of cancer cells will make it easily break through the mechanical pressure and immune monitoring, and enter the peripheral vein to become circulating tumor cells (CTCs).^31^ Peripheral blood neutrophils and platelets can inhibit NK cells by secreting cytokines such as IL‐1 and vascular endothelial growth factor‐A, and promote immune escape and drug resistance of CTCs.^32^, ^33^, ^34^, ^35^ Okabe et al. ^36^ found that CTCs are an important factor related to the prognosis of patients with advanced stage GC, and the probability of detection of CTCs in diffuse type GC is twice as high as that in intestinal type GC. Meanwhile, NK cells and macrophages in peripheral blood play a role in monitoring CTCs. This study found that the percentage of lymphocytes increased in patients with advanced diffuse/mixed GC, which may be related to the increase of CTCs, but whether this increase is the killing effect of CTCs needs further study. In addition, peripheral blood immune cells not only play an inhibitory role in the process of distant metastasis, but also play a promoting role. This functional difference is closely related to different tumor stages. CD45RA‐CCR7‐ regulatory T cells can inhibit the activity of CD8+ T cells and promote the immune escape of cancer cells in advanced GC.^37^, ^38^ Therefore, exploring the level and function of immune cells according to tumor stage and Lauren classification will improve the clinical application.

The inflammatory indexes SII and PLR calculated by circulating immune cells have been proved to be clinical markers for predicting the prognosis of GC patients, which can be used to identify high‐risk patients and guide treatment. Through ROC analysis, we found that the AUC of SII was higher in patients with intestinal type GC, which was an independent prognostic factor. This also showed that peripheral blood neutrophils, lymphocytes, and platelets play an important role in the external circulation immunity of patients with intestinal type GC. The AUC of PLR was higher in patients with diffuse/mixed GC, which was an independent prognostic factor. It is further suggested that the immune cell subsets that play an important role in the external circulation immunity of different Lauren classification are different. In addition, SII was negatively correlated with BMI and positively correlated with tumor diameter and PLR was positively correlated with tumor diameter, which was similar to the current study. Lee et al. ^39^ found that for patients undergoing radical gastrectomy, overweight or mild to moderate obesity (23 kg/m2≤BMI<30 kg/m2) was associated with better survival probability. Patients with higher BMI had lower invasive ability of tumor cells and the prognosis of overweight patients can be improved if they reduce their weight and improve their nutritional status.^40^, ^41^ Similarly, the risk of lymph node metastasis increases significantly with tumor diameter. Kim et al. ^42^ constructed a nomogram combining tumor diameter with age, vascular invasion, and T stage to evaluate the risk of lymph node metastasis in patients with early GC. We found that preoperative SII and PLR can not only evaluate the prognosis of patients with intestinal type and diffuse/mixed type GC, but also reflect the nutritional status of patients. The combination of SII and PLR can also evaluate the risk of lymph node metastasis, which can provide more treatment information before surgery.

We found that patients with intestinal type GC have better postoperative chemotherapy sensitivity, and patients with high levels of SII have better chemotherapy efficacy, which confirms the study of Paula et al..^8^ The sensitivity of tumor cells to chemotherapeutic drugs depends not only on the cancer cells, but also on the immune status of the tumor microenvironment. Wang et al. ^43^ found that patients with neutropenia after chemotherapy have better survival rate. Diffuse GC cells are rich in mucus and lysosomes and lack ribosome structure, which makes them insensitive to a variety of chemotherapy drugs. Studies have shown that CYP2A6 ^44^ may affect the therapeutic effect of patients with intestinal type GC due to serious complications even if they receive postoperative chemotherapy. Therefore, it is necessary to study further whether patients with diffuse GC can improve postoperative survival by targeted therapy.

In clinical practice, medical experts have gradually found that TNM stage based on macroscopic tumor anatomy provides effective but incomplete information for treatment. In patients at the same stage, the prognosis and sensitivity to adjuvant therapy often show significant individual differences. More studies suggest that tumor immunity can play a complementary role.^45^, ^46^ For example, adaptive immune response can predict the recurrence of cancer patients based on the recognition of T cells for recurrent tumor cell surface antigens.^47^ Liu et al. y^48^ constructed a nomogram based on the system prediction score, tumor location, and TNM stage to evaluate the prognosis of patients with stage II or III GC. Therefore, this kind of model constructed by combining immune markers with clinicopathological features has the advantage of phenotype of disease heterogeneity, and can evaluate the prognosis of patients more accurately. Our study analyzed the sensitivity of patients with different Lauren subtypes to inflammation biomarkers. According to multivariate analysis, SII, pTNM stage, and postoperative chemotherapy were independent factors related to the prognosis of patients with intestinal type GC. PLR and pTNM stage were independent factors related to the prognosis of patients with diffuse/mixed GC. Then, we constructed nomogram models to predict the prognosis of patients with different Lauren classification. ROC analysis showed that the AUC of predicting 3‐year and 5‐year prognosis of patients with intestinal type GC were 0.796 and 0.807, the sensitivity were 72.2% and 75.5%, and the specificity were 79.1% and 76.2%. The AUC of predicting 3‐year and 5‐year prognosis of diffuse/mixed type patients were 0.791 and 0.788, the sensitivity were 83.1% and 81.7%, and the specificity were 63.0% and 66.5%. The prediction model constructed by inflammatory biomarkers and clinicopathological features can effectively evaluate the prognosis of patients with different Lauren classification, which is worthy of further validation and promotion in clinical practice.

As a retrospective study, there were still some limitations. First, although PSM score was used to deal with the differences between the two groups, there may still be bias factors influencing the study. Second, this study only focused on an Asian population in a single center. Whether the results are widely applicable to white and black populations needs to be further studied by expanding the sample size.

## 5 CONCLUSIONS

Peripheral circulating immune cells had different distribution based on Lauren classification. Intestinal type GC patients (*p* < 0.05) had a lower percentage of neutrophils in stage I, higher percentage of neutrophils and higher platelet count in stage Ⅲ (*p* < 0.05). SII was an independent prognostic factor for patients with intestinal type GC, which could be combined with postoperative chemotherapy and pTNM stage to construct a nomogram to predict prognosis. PLR was an independent prognostic factor for patients with diffuse/mixed GC, which could be combined with pTNM stage to construct a nomogram to predict prognosis.

## CONFLICT OF INTEREST

The authors declare that they have no conflict of interest.

## AUTHOR CONTRIBUTION

Xin Yin and Tianyi Fang designed and conceived this project, and they contributed equally to this work. Xin Yin, Tianyi Fang, and Yimin Wang interpreted and analyzed the data. Yingwei Xue revised the manuscript for important intellectual content. Xin Yin, Tianyi Fang, Yimin Wang, Yufei Wang, Daoxu Zhang, and Chunfeng Li participated in the patient information collection.

## ETHICAL APPROVAL

All programs followed were according to the ethical standards of the Human Subjects Responsibility Committee (institutions and countries), as well as the 1964 Helsinki Declaration and subsequent editions. This research was approved by the Ethics Committee of the Harbin Medical University Cancer Hospital (Approval Number: SHGC‐1029).

## Data Availability

Patients’ data were saved in the Gastric Cancer Information Management System v1.2 of Harbin Medical University Cancer Hospital (Copyright No.2013SR087424, *http*:www.sgihmu.com).
